# Arginine-Coated Nanoglobules for the Nasal Delivery of Insulin

**DOI:** 10.3390/pharmaceutics15020353

**Published:** 2023-01-20

**Authors:** Atanu Das, Richa Vartak, Md Asrarul Islam, Sunil Kumar, Jun Shao, Ketan Patel

**Affiliations:** College of Pharmacy and Health Sciences, St. John’s University, Queens, New York, NY 11439, USA

**Keywords:** insulin, nanoemulsion, nasal delivery, permeability enhancement, lauryl arginine ethyl ester

## Abstract

Multiple daily injections via subcutaneous route are the primary modes of insulin delivery for patients with Diabetes Mellitus. While this process is invasive, painful and may cause patients to develop lipohypertrophy at injection site, the perception of fear surrounding this process causes patients to delay in initiation and remain persistent with insulin therapy over time. Moreover, poor glycemic control may often lead to acute complications, such as severe hypoglycemia and nocturnal hypoglycemia, especially in older patients with diabetes. To address the imperative need for a patient-convenient non-invasive insulin therapy, an insulin-loaded arginine-coated self-emulsifying nanoglobule system (INS-LANano) was developed for nasal delivery of insulin with a biodegradable cationic surfactant—Lauroyl Ethyl Arginate (LAE). Incorporation of LAE resulted in formation of positively charged nanoglobules with L-arginine oriented on the surface. LANano enabled binding of insulin molecules on the surface of nanoglobules via an electrostatic interaction between negatively charged α-helix and LAE molecules at physiological pH. INS-LANano showed a hydrodynamic diameter of 23.38 nm with a surface charge of +0.118 mV. The binding efficiency of insulin on LANano globules was confirmed by zeta potential, circular dichroism (CD) spectroscopy and centrifugal ultrafiltration studies. The attachment of insulin with permeation-enhancing nanoglobules demonstrated significantly higher in vitro permeability of insulin of 15.2% compared to insulin solution across human airway epithelial cell (Calu-3) monolayer. Upon intranasal administration of INS-LANano to diabetic rats at 2 IU/kg insulin dose, a rapid absorption of insulin with significantly higher Cmax of 14.3 mU/L and relative bioavailability (BA) of 23.3% was observed. Therefore, the INS-LANano formulation significant translational potential for intranasal delivery of insulin

## 1. Introduction

Diabetes Mellitus (DM) is characterized by chronic elevation of blood glucose levels due to insufficient insulin secretion from pancreatic β cells or the development of resistance in peripheral tissues to insulin or both. Insulin supplement remains the core therapy for the management of DM for most patients with type 2 DM and all patients with type 1 DM. Although much progress has been made in the development of patient-convenient insulin therapy over the past 100 years, multiple injections of insulin by subcutaneous route remain the primary mode of insulin delivery [1]. However, it is inconvenient for patients to administer it daily since it is invasive in nature and chronic administration may lead to potential cutaneous problems, such as lipohypertrophy, characterized by lobules of large adipocytes due to fat and protein synthesis surrounding the injection area [2]. Investigations on psychological barriers against invasive insulin therapy reveal that “fear of injection” has multiple forms that include daily painful process, concerns about technical skills, infliction of self-harm and perception of the social embarrassment of using at public places, all of which cause patients to delay the initiation and maintain persistency with insulin therapy over time [3]. While varieties of commercial basal and prandial insulin injections are available, glycemic variability remains a major issue that may often lead to acute complications, such as severe hypoglycemia and nocturnal hypoglycemia in older patients and diabetic ketoacidosis in younger patients. The only commercial non-invasive insulin formulation Afrezza^®^, a rapid-acting insulin formulation administered via the inhalation route, has been reported to cause a decline in lung function over time, cough, irritation and acute bronchospasm in asthmatics [4]. Therefore, the need for insulin therapy for diabetic patients to achieve good metabolic control, convenient self-administration, psychological well-being and, ultimately, a good quality of life is not fulfilled yet.

Insulin is a hydrophilic macromolecule of 5808 Da molecular weight and is composed of 51-amino acids divided into two chains, Chain A: 21 amino acids and Chain B: 30 amino acids, where both chains are linked together by two inter-chain disulfide bonds with an additional intra-chain disulfide bond formed within the A chain. The isoelectric point of insulin is 5.3 [5]. Non-invasive delivery of insulin is challenging since it is difficult for insulin to permeate through the lipid membrane by itself due to its hydrophilic nature. It is well known that the main absorption route for this type of drug is paracellular; however, the tight junction (TJ) between the cells is the main obstruction for its paracellular absorption due to its macromolecular size. Many research activities have been conducted on the delivery of proteins and macromolecules using various permeation enhancers, e.g., surfactants, bile salts, cyclodextrins, cell-penetrating peptides, etc. to improve their paracellular absorption [6]. The nasal route has great potential for the delivery of administered drugs due to its relatively thin and porous epithelium with abundant vasculature structure that leads to higher permeability as compared to buccal and transdermal routes. Unlike the oral or intestinal route, it is devoid of extensive enzymatic degradation and hepatic first-pass metabolism of the administered drug [1]. In addition, a recent study suggests that intranasally administered insulin acts as a neuroprotective agent for ischemic stroke, subarachnoid hemorrhage and traumatic brain injury due to its ability to bypass the blood–brain barrier to distribute into the cerebrospinal fluid at a higher concentration [7].

Self-emulsified nanoemulsion (SEN) is a thermodynamically stable system that spontaneously forms upon dilution of the preconcentrate, which is a combination of surfactant(s) and lipid(s) with or without cosolvents [8,9]. SEN has the potential to offer enhanced permeability of the loaded drug across lipidic membranes due to its nanosized lipid globules surrounded by surfactant and its ability to permeate through mucus membranes. Lauroyl ethyl arginate (LAE) is an amino acid-based cationic surfactant that is a derivative of a hydrophilic moiety- L-arginine, conjugated with a lipophilic part- lauric acid. Previously, LAE was used in the nanoemulsion system, where LAE-loaded preconcentrate was spontaneously self-emulsified to produce LAE-anchored nanoglobules that were found to enhance the permeability of small drug molecules [10]. In this research, LAE was explored as an insulin-binding agent and permeation enhancer.

The objectives of this research are to (I) develop and characterize an insulin-loaded arginine-coated self-emulsifying nanoglobule system (Ins-LANano) (II) investigate the in vitro permeation of insulin from Ins-LANano through a human airway epithelial cell (Calu-3) monolayer and, finally, (III) investigate the absorption and bioavailability profile of insulin upon administration of intranasal Ins-LANano in streptozotocin-induced diabetic rats.

## 2. Materials and Methods

### 2.1. Materials

Human insulin (27.5 IU/mg), Lauroyl ethyl arginate, Kolliphor^®^ RH 40, Centriprep^®^ centrifugal filter unit and Bovine serum albumin fraction V were purchased from Sigma-Aldrich, Co. (St. Louis, MO, USA). Capmul^®^ MCM NF and Captex^®^ 8000 were gifted from Abitec Corporation (Janesville, WI, USA). Human lung cancer cell line (Calu-3) was purchased ATCC^®^ (Rockville, MD, USA) and Thiazolyl blue tetrazolium bromide, 98% was procured from Acros Organics (Morris Plains, NJ, USA). Transwell inserts were purchased from Corning (Oneonta, NY, USA). Fetal Bovine Serum was purchased from Atlanta Biologicals (Flowery Branch, GA, USA). AimStrip^®^ Plus glucometer and glucose strips were procured from Germaine Laboratories, Inc (San-Antonio, TX, USA). Human Insulin ELISA Kit was purchased from Crystal Chem (Elk Grove Village, IL, USA). Acetonitrile, methanol, trifluoroacetic acid (TFA) and citrate buffer solution (0.5 M, pH 4.5) were sourced from Fisher Scientific. Dulbecco’s Phosphate-Buffer Saline, pH 7.0 was purchased from Hyclone (Logan, UT, USA). Streptozotocin was procured from Adipogen^®^ Life Sciences (San Diego, CA, USA). All the purchased components were of analytical grade.

### 2.2. HPLC Method

HPLC analysis of insulin was conducted using an Agilent 1260 system with a Gradient HPLC pump, an automated sample injector and a UV detector. Separation was obtained using a Symmetry^®^ C18 column (4.6 × 150 mm) having a pore size of 100 Å and particle size of 5 µm. Mobile phase A consisted of deionized water with 0.1% (*v*/*v*) Trifluoracetic acid and Mobile Phase B consisted of 90% Acetonitrile and 10% Deionized water with 0.1% TFA. The flow rate of the mobile phase was 1 mL/min, the injection volume was 20 µL, the detection wavelength was 220 nm and the total run time for each sample was 45 min. The retention time for insulin was 16.5 ± 0.2 min and the total peak area was used to estimate the concentration of insulin. The method was linear in the range of 10–200 µg/mL (R2 = 0.999)

### 2.3. Preparation of Arginine-Coated Self-Emulsifying Nanoglobule System (Lanano)

To prepare LANano, LAE was added to a preconcentrate consisting of a medium chain monoglyceride (MCM): Capmul MCM, a medium chain triglyceride (MCT): Captex 8000; and a surfactant: Kolliphor^®^ RH 40 at a weight ratio of 2:3:4. At first, the lipids and surfactants were mixed in an orbital shaker incubator for 1 h at 300 rpm and 37 °C temperature to prepare the clear homogenous pre-concentrate. LAE was added to the above preconcentrate and the mixture was heated in a water bath at about 50 °C to melt and dissolve LAE in the pre-concentrate. Then, LAE-loaded preconcentrate was diluted with an aqueous medium with gentle shaking to prepare the LANano. A stock solution of insulin (INS-SOL) at 1 mg/mL concentration was prepared and added to LANano to form INS-LANano with the required concentration of insulin in the formulation.

### 2.4. Size and Zeta Potential

The globule size, polydispersity index (PDI) and zeta potential of different formulations were measured by dynamic light scattering technique using a Zetasizer^®^ Nano ZS particle size analyzer (Malvern Instrument Inc., MA, USA). Samples were directly loaded into cuvettes and analyzed at 25 °C with a scattering angle of 173°. The hydrodynamic diameters of the globules were automatically calculated with the instrument’s software based on the analysis of the autocorrelation function [11].

### 2.5. Circular Dichroism (Cd) Spectroscopy

The CD spectrophotometer: Jasco J-1500 (JASCO North America, Easton, MD, USA) was used to obtain the CD spectra of insulin from an average of three scans in the far-UV region with the following experimental conditions: Wavelength: 190–260 nm; Temperature: 20 °C; Cell Pathlength: 0.1 cm; Step Size: 0.5 nm; Bandwidth: 1 nm; and Scanning speed: 100 nm/min. Different INS-LANanos were prepared and diluted to insulin concentration of 100 µg/mL and LAE at 100, 250, 500, 750 and 1000 µg/mL concentration to achieve insulin to LAE ratios of 1:1, 1:2.5, 1:5, 1:7.5 and 1:10. Insulin solution in Phosphate-Buffer Saline (PBS) at 100 µg/mL concentration served as the control formulation to compare the CD spectra and respective α-helix content of insulin from different formulations. CD spectra of the appropriate backgrounds were recorded and subtracted from the spectra of respective proteins.

CD intensity was expressed as mean residue ellipticity, calculated as per Equation (1):

(1) $$\Theta \mathrm{mre}=\frac{\mathrm{MRW}.[\Theta ]\lambda }{10.l.c}$$

where [Θ]mre = mean residue ellipticity (unit: deg.cm2.dmol-1); MRW= mean residue weight of insulin (115.87 Da); [Θ]λ = observed ellipticity at wavelength λ (unit: mdeg); l = optical path length of cell (unit: cm); and c = insulin concentration in g/L [12]. The α-helix content of insulin was estimated by a web server, BestSel: http://bestsel.elte.hu/index.php [13].

### 2.6. Binding Efficiency

The binding between insulin and LAE on LANano globules was investigated by centrifugal ultrafiltration method using Centriprep^®^ centrifugal filter unit which consists of a donor and receiver chamber separated by a membrane filter with a molecular weight cut-off of 50 kDa. INS-LANano (insulin to LAE ratio of 1:2.5) was added to the donor chamber of the filter unit and centrifuged at 3000× *g* for 15 min to separate the aqueous medium in the receiver chamber. The concentration of insulin in INS-LANano in the donor chamber before centrifugation and in the aqueous medium in the receiver chamber after centrifugation was analyzed by HPLC to determine the concentration of initially added insulin and recovered insulin, respectively. The Insulin solution (INS-SOL) and insulin-loaded self-emulsifying nanoglobule system (INS-SEN) at a 100 µg/mL concentration of insulin served as controls to determine the loss of initially added insulin due to membrane adsorption.

### 2.7. Cytocompatibiliy Study

Calu-3 cells were cultured in T-75 flasks in a culture medium containing EMEM (with phenol red), 10% heat-inactivated FBS and 1% penicillin-streptomycin solution. Cells were cultured in a humidified incubator at 37 °C with 5% CO_2_. Cells were split using trypsin (0.25%) with 0.53 mM EDTA solution and subcultured when they reached 90% confluency.

The cytocompatibility assay of nano-formulation was assessed using Calu-3 cell line using Crystal Violet assay. Briefly, 96-well plates with 20,000 cells per well were seeded and allowed to form a monolayer overnight at 37 °C and 5% CO_2_. Medium from each well was withdrawn and replaced with an equal volume of the different treatments having the range of concentration from 0.01–2.0 mg/mL and further incubated for 3-h. After 3-h, cells were washed with PBS, fixed using 4% *v*/*v* glutaraldehyde and stained with 0.5% *w*/*v* crystal violet. Excess crystal violet was washed using HPLC-grade water and was air-dried overnight. With an inverted microscope (Evos XL core imaging system; Thermo Fisher Scientific, Waltham, MA, USA), images were captured at ×20 magnification. Cells were lysed with 0.5% (*w*/*w*) SLS and the absorbance was read at 560 nm for cell viability [14].

### 2.8. Permeability Study

INS-LANano was evaluated for insulin permeability across Calu-3 cell monolayer and compared with INS-SEN and INS-SOL. HBSS with 1% bovine serum albumin (BSA) (pH = 7.4) was used as the medium for the transport experiment. BSA has been reported to reduce the surface binding of peptides and proteins to the surface of plastic and glassware by non-specific interaction that may unexpectedly lead to inaccurate estimations of total peptide content [15]. The LANano and SEN were prepared by diluting the preconcentrates in HBSS with 1% BSA. The concentration of LAE in INS-LANano was 0.1 mg/mL. The formulations were spiked with a stock solution of insulin to achieve a 30 µg/mL of the final concentration of Insulin. The pH of all formulations was adjusted to 6 to resemble the nasal pH [16].

Briefly, cells were seeded in 12-well Transwell^®^ plates with a polycarbonate insert (1.38 cm^2^, 8.0 μm). Each well was filled with 1.5 mL of medium in the basolateral region and 0.5 mL in the apical region and the medium was replaced each alternate day. Transepithelial electrical resistance (TEER) was measured each day following medium replacement using a Millicell ERS-2 (Millipore Sigma, Jaffrey, NH, USA) device probe until a TEER value of 600 ohm-cm^2^ was achieved, which was indicative of monolayer formation. Upon the formation of the Calu-3 cell monolayer, the culture medium from the apical and basolateral regions was removed. The cells were washed and equilibrated in HBSS with 1% BSA for 30 min. A 0.5 mL of each formulation was added to the apical sides of the wells and 1.5 mL of HBSS with 1% BSA (at pH 7.4) was added to the basolateral side. The Transwell^®^ plates were incubated at 37 °C on a mechanically controlled shaker at 30 rpm for 2 h to minimize stagnation of the boundary layer. After 2 h, samples were collected from the basolateral side and replaced with a fresh culture medium. Samples were analyzed for determination of insulin concentration by an ultrasensitive human-insulin ELISA kit following the procedure as detailed by the manufacturer and the absorbance of the colorimetric endpoint of the test is measured on a GloMax^®^ Discover microplate reader. The concentration of insulin post 2 h of transport from different formulations were compared with the initial concentration of insulin to determine the permeability of respective formulations. TEER values after 2 h and 24 h were recorded to evaluate the effect of formulations on monolayer integrity and toxicity.

### 2.9. In Vivo Study

An in vivo pharmacokinetic (PK) study was conducted on male Sprague Dawley rats. The rats were induced with diabetes with Streptozotocin (STZ) before use for the experiment. Briefly, rats were kept on fasting with water ad libitum for 12–14 h before the diabetic induction. STZ solution in 0.1 M ice-cold citrate buffer was freshly prepared and immediately injected into the fasted rats at 55 mg/kg body weight via the intraperitoneal route. Rats were allowed to have food and water ad libitum. After 48 h of STZ injection, the fed state blood glucose was measured and rats with blood glucose levels higher than 250 mg/dL were considered diabetic and were used in the PK study [17].

Streptozotocin-induced diabetic rats (250–290 g) were randomly divided into four treatment groups, such as, INS-LANano (intranasal), INS-SEN (intranasal), INS-SOL (intranasal) and INS-SOL (subcutaneous). Rats fasted with water ad libitum for 12–14 h before the experiment. Rats were also kept in non-anesthetized conditions. The intranasal dose of human insulin from INS-LANano, INS-SEN and INS-SOL was 2 IU/kg and the subcutaneous dose of insulin from INS-SOL was 1 IU/kg. The nasal administration was conducted using a micropipette and tips. Following administration of the formulations, blood samples were collected by tail tipping method into heparin-coated microcentrifuge tubes for determination of absorbed insulin concentration in plasma from administered insulin. Lidocaine-containing gel was applied to the tails of rats during tail tipping. After the final sampling at 120 min, the animals were euthanized by asphyxiation in a carbon dioxide chamber. The blood samples were centrifuged at 10,000× g for 5 min to separate the plasma. Plasma samples were collected and stored at −20 °C until further analysis. Plasma concentration of insulin was determined by an ultrasensitive human-insulin ELISA kit following the procedure as detailed by the manufacturer.

Standard non-compartmental analysis was performed using Phoenix WinNonlin 8.3 to determine the PK parameters. The area under the insulin concentration-time curve for each formulation was calculated for 0–120 min using the trapezoid rule. The subcutaneous group served as the control to calculate the relative bioavailability (BA) of intranasally administered insulin for subcutaneously administered insulin.

### 2.10. Statistical Analysis

The data are expressed as the mean ± standard deviation or standard error of the mean. Student’s *t*-test for binding efficiency study and one-way analysis of variance (ANOVA) followed by Tukey’s multiple comparisons post hoc tests for permeability and pharmacokinetic studies were used to determine the significant difference of data between different groups. The software used for analysis was GraphPad Prism version 9.4.0 (GraphPad Software, San Diego, CA, USA).

## 3. Results

### 3.1. Particle Size and Zeta Potential

**Figure 1:**
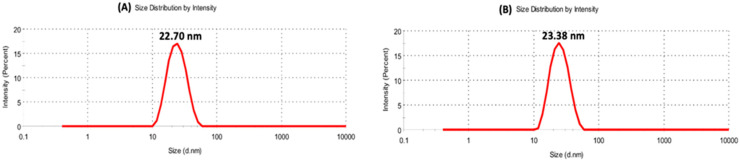
DLS Particle size of LANano (**A**) and INS-LANano (**B**).

Table 1 shows the zeta potential and size of blank SEN and LANano in a water medium. SEN showed close to neutral zeta potential while incorporation of LAE into SEN to form LANano resulted in a significant enhancement in zeta potential. It confirmed that LAE is oriented in a way that L-arginine molecule (guanidino group) is exposed on the surface. At the same time, surface active property of LAE resulted in a drastic reduction in particle size of nanoglobules. The presence of LAE on the lipid-water interface facilitated the self-emulsification of lipids in water and, consequently, resulted in the reduction in globule size of LANano to that of SEN. When PBS was used as the medium to prepare LANano, a decrease in surface charge and an increase in globule size (Figure 1A) was observed. Buffer components in PBS caused partial neutralization of the overall positive charge of LAE.

Furthermore, batches were prepared using different ratio of human insulin. Table 2 shows zeta potential and size for different INS-LANano, where the ratio of LAE to insulin was in the range of 1:0.1 to 1:1. Particle size remained unaffected on addition of insulin (Figure 1). A gradual decrease in positive charge of INS-LANano was observed with the increase in insulin concentration, which may be due to the electrostatic interaction between negatively charged insulin and positively charged LAE that causes insulin to bind on the surface of LANano. No significant globule size and PDI change was observed at different LAE to insulin ratios. No aggregation was observed at higher insulin concentrations.

### 3.2. Circular Dichroism (Cd) Spectroscopy Study

**Figure 2:**
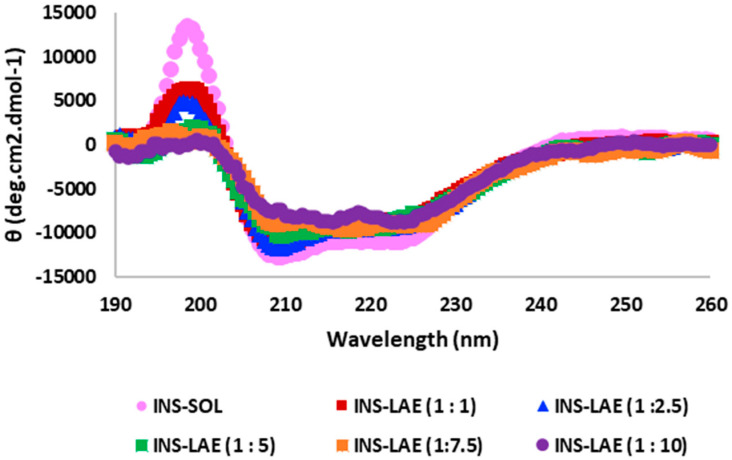
Far-UV CD spectra of insulin from different formulations.

The CD spectroscopy was used to investigate the structural integrity of insulin in Ins-LANano and the possible interaction of insulin with LAE. According to previous reports, the CD spectrum of insulin solution displays one peak or positive extremum at 196 nm and two valleys or negative extremum at 208 nm and 222 nm, which are indicative of its predominantly α-helical secondary structure and the analysis of secondary structural content revealed that the estimated α-helix content is approximately 42% [12,18,19]. Figure 2 shows the CD spectra of insulin from control and test formulations.

Table 3 shows the wavelengths where the peak/valley appeared in spectra and the α-helix contents for respective formulations. The peak/valley wavelengths are in close agreement with the published articles; however, as observed from the patterns of spectra, the mean residue ellipticities gradually decreased for 196 nm and increased for 208 nm and 222 nm as the ratios of insulin to LAE increased in respective INS-LANano. Additionally, the α-helix content of insulin decreased with an increase in LAE concentration. The trend of change in spectra along with their mean residue ellipticities and respective α-helix contents may be due to the interaction of insulin with LAE.

### 3.3. Binding Efficiency Study

The centrifugal ultrafiltration of INS-LANano through a 50 kDa membrane was performed to prove the binding interaction of Insulin with LAE on LANano globules. The initial insulin concentration in INS-LANano formulation before centrifugation was 100.5 ± 4.0% (*n* = 3). The recovery of insulin in the receiver chamber, as shown in Table 4, indicated that retention of insulin in the donor chamber due to non-specific adsorption of insulin to the membrane filter during the centrifugation and hindrance imparted by nanoglobules are about 8.5% and 13.6%, respectively. Nanoglobules are relatively larger as compared to the pores of membrane ultrafilter and, therefore, retain in the donor chamber. Similarly, insulin molecules that are bound to the surface of LANano globules are not able to pass through the membrane which is evident from the lower recovery of insulin corresponding to 53.6 ± 0.1%, from INS-LANano formulations in the receiver chamber as compared to the recovery of insulin from INS-SEN alone. The leakage of around 47% suggests that the binding between Arginine and insulin on the LANano surface is not very strong. Centrifugal force might result in the separation of the Insulin-LAE complex from the globule.

### 3.4. Cytocompatibility Study

**Figure 3:**
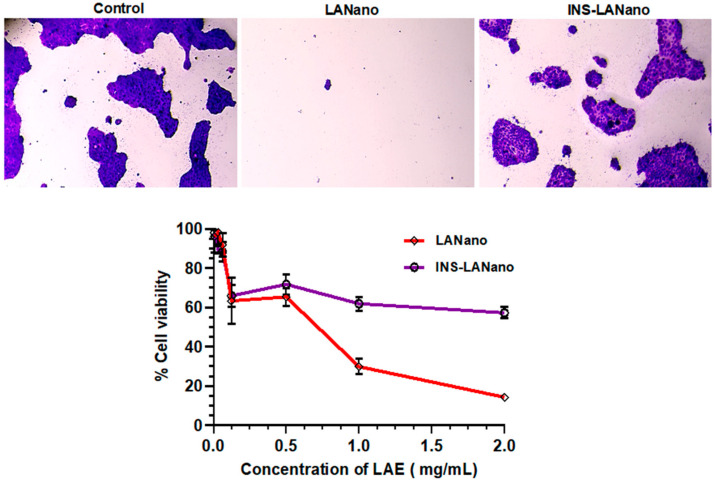
Cytocompatibility of formulations on Calu-3 monolayer.

Figure 3 shows that after 3 h, INS-LANano showed higher cell viability than LANano. The binding of Insulin to LAE on INS-LANano globules reduced the positive charge of LANano globules and, thereby, improve the cytocompatibility of formulation.

### 3.5. Permeability Study

**Figure 4:**
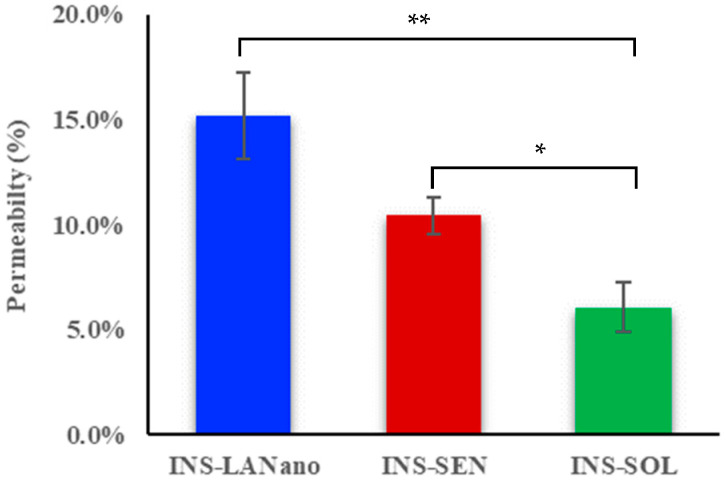
Permeability of insulin from different formulations across Calu-3 monolayer. Mean ± SD. *n* = 3–4. * = significantly higher than INS-SOL. ** = significantly higher than INS-SEN. (*p* < 0.01).

In vitro permeation of insulin by INS-LANano was determined and compared with INS-SEN and INS-SOL following 2 h of transport study across a Calu-3 cell monolayer. The result of the permeability experiment is illustrated in Figure 4. Plain INS-SOL showed a 6.1% insulin permeation while significantly higher insulin permeability of 10.4% by INS-SEN was observed. A 15.2 ± 2.1% of insulin permeability was achieved by INS-LANano, which is significantly higher as compared to that by INS-SEN and INS-SOL. A 2.5-fold improvement in insulin permeation from INS-LANano was observed when compared to INS-SOL. Therefore, INS-LANano appeared to be a potential formulation to proceed with for an in vivo experiment.

To evaluate the integrity of monolayers, the TEER was measured before the experiment and was found to be approximately 600 Ω.cm^2^. A marginal reduction of 15% in TEER values at the end of 2 h was observed in post-exposure of INS-LANano in comparison to the control. No significant reduction in TEER value was observed for the INS-SOL group. However, after 24 h, the TEER value for the INS-LANano group was found to be similar to the initial TEER value that confirmed the reversible opening of the TJ and LANano is not damaging the monolayer integrity.

### 3.6. In Vivo Study

**Figure 5:**
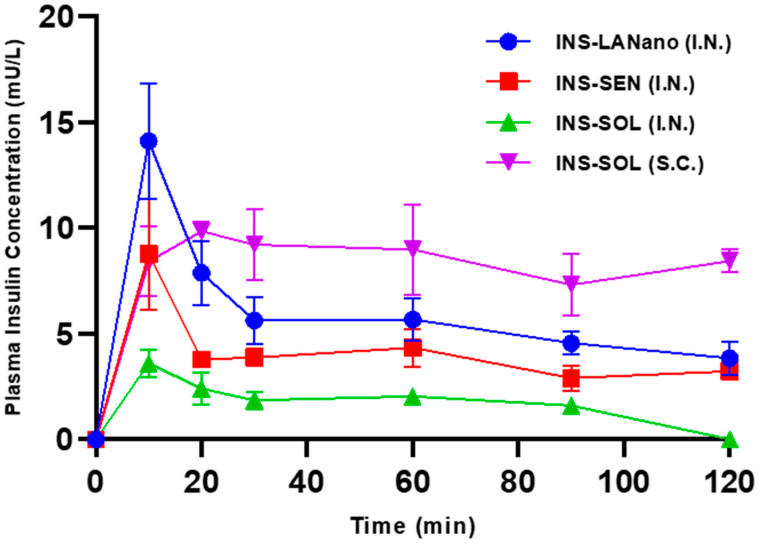
Plasma insulin concentration-Time profile in diabetic rats following 1 IU/kg subcutaneous dose and 2 IU/kg intranasal dose.

The in vivo study was conducted with the animal use protocol approved by St. John’s University IACUC committee with protocol number 2002. In Figure 5, the plasma insulin concentration-time curve following intranasal administration of INS-LANano, INS-SEN and INS-SOL and subcutaneous administration of INS-SOL to diabetic rats is shown. In Table 5, the pharmacokinetic parameters of insulin are summarized that are derived from the insulin concentration-time curve. All formulations delivered via nasal route at 2 IU/kg showed rapid absorption of insulin and reached their maximum concentration in plasma within 15 min after administration. INS-SOL (I.N.) resulted in limited absorption of insulin. Though there was no significant difference among Tmax values, the BA of insulin from INS-LANano (I.N.) was significantly higher than that from INS-SEN (I.N.) and INS-SOL (I.N.) and the Cmax of insulin from INS-LANano (I.N.) was significantly higher than that from INS-SOL (I.N.). Importantly, all intranasal formulations showed very short Tmax as compared to subcutaneous solution. The in vivo absorption profile of insulin is in correlation with in vitro permeability results. A comparative analysis of our developed INS-LANano with the marketed formulation Afrezza^®^ has been provided (Table 6).

## 4. Discussion

**Figure 6:**
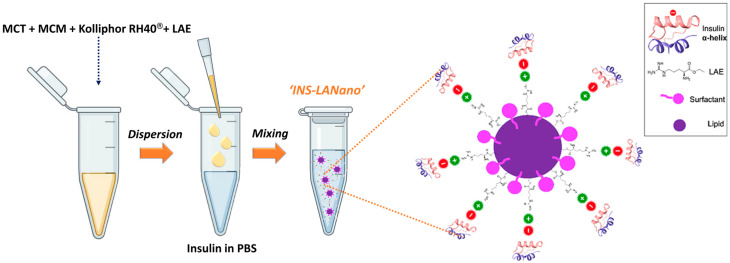
Schematic representation depicting fabrication of insulin-loaded arginine-coated self-emulsifying nanoglobules system.

We have explored a biodegradable, cationic surfactant to facilitate the binding between nanoglobules and insulin to improve the transport of insulin across nasal epithelium. LAE acts as an antimicrobial agent against foodborne bacteria and was previously reported as a safe molecule for consumer products since it undergoes rapid metabolism to produce naturally occurring dietary components: lauric acid and arginine. Additionally, LAE is approved by the Food and Drug Administration (FDA) and the European Food Safety Agency (EFSA) as a preservative in the packaging of food materials. In the search of a safe preservative for the intranasal insulin formulation, INS-LANano was developed and evaluated for its efficacy to enhance the permeability of insulin across nasal membranes in vitro and in vivo. LAE is an ester form of L-arginine amino acid that carries positive charges in solution at neutral and physiological pH. Upon its incorporation into LANano globules, LAE resides on the surface of nano-globules that eventually generated an overall positive charge on the surface of LANano globules. The isoelectric point of insulin is 5.3 and, therefore, in a solution with a pH above its isoelectric point, insulin carries a net negative charge. Therefore, in the INS-LANano system, insulin tends to bind with LAE on the nanoglobule surface via electrostatic interaction. This phenomenon is illustrated in Figure 6. A gradual increase in insulin concentration in the system causes the overall charge of the system to turn from positive to negative which indicates an increasing trend of binding between insulin and LANano.

Zeta potential, CD and ultrafiltration method confirmed that insulin was bound to the nanoglobules. We hypothesized that insulin binds to positively charged LA-nano using negatively charged α-helix with two glutamic acid residues. The CD is mostly used to probe the secondary structure of a protein and its binding interaction with ligands. The CD spectrum of insulin from INS-LANano showed the characteristic shape of an α-helical protein with its peak and valley region. However, the gradual changes in mean residue ellipticities at peak and valley wavelengths along with the gradual decrease in helix content with an increase in insulin to LAE ratio indicates the variable degree of binding between insulin and LAE. The characteristic shape of the spectrum of an α-helical protein emerges from the coupling of π → π* transition moments in each amide chromophore in the protein. Many ligands that bind to a macromolecule in biological systems fluster its secondary structure by perturbing its electronic transitions to some extent [22]. Miguel et al. observed that the shape of the spectrum and the α-helix content of a signal peptide differs dramatically from 45% to 25% in presence of 0.5% SDS [23].

The binding of insulin with LANano globules was also evident from the comparatively lower recovery of insulin in the receiver chamber from INS-LANano than that of INS-SEN. The centrifugal ultrafiltration method was previously used by Darshana et al. to determine the complexation of insulin with phosphatidylcholine and the consequent entrapment of insulin in preconcentrate globules [24]. The intranasal aqueous formulation contains a preservative for its long-term storage. Cationic surfactants can be used as antimicrobial agents because of their chemical nature and ability to disintegrate the bacterial cell membrane at very low concentrations. This change produces an altered membrane potential that affects cell permeability and consequently leads to bacterial cell death [10]. However, a cationic surfactant, such as Benzalkonium chloride, was reported to cause side effects, such as an increased risk in developing severe hyponatremia and seizures upon chronic use. An investigation of the cause of these side effects revealed that benzalkonium chloride affects the integrity of the nasal mucosa and causes uncontrolled drug permeation and bioavailability [25]. Thus, LAE based system could be superior to Benzalkonium chloride-based permeation enhancement.

One of the major challenges of intranasal delivery of insulin is the possibility of degradation of insulin by proteolytic enzyme that may limit the intranasal absorption of insulin. In a study to investigate the effect, a clinically relevant amount of insulin was incubated with freshly collected human nasal wash (to imitates the exposure of insulin to local enzymes present in the nasal cavity), isolated enzyme from pig nasal mucosal tissue, leucine aminopeptidase and microsomal aminopeptidase (to imitates the exposure taking place during the passage of insulin through the membrane into the circulation). After 3 h of incubation at 37 °C, the recoveries were 96%, 98%, 99% and 99% for human nasal wash, pig nasal mucosal tissue, leucine aminopeptidase and microsomal aminopeptidase, respectively. It was concluded that the enzymatic degradation was not observed to be a limiting factor for intranasal application of Insulin [26]. On similar lines, it can be ascertained that there will not be any significant degradation of insulin from INS-LANano formulation due to proteolytic enzymes.

Calu-3 is a human airway adenocarcinoma epithelial cell that forms a monolayer with functional tight junctions, desmosomes and zonular adherence. The mucus layer secretes mucin that consists of mucin genes 5 (MUC5), the major mucins present in human healthy airway secretions. Therefore, the Calu-3 cell monolayer mimics the human nasal epithelium and has been widely used as a cell culture model for nasal drug absorption [27,28,29]. Therefore, it was selected for the current study to evaluate the cytocompatibility and permeation enhancement ability of the developed formulation. The cytocompatibility assay was conducted for 3 h considering the shorter retention time of formulations in the nasal cavity. After the 3 h period, comparatively less toxicity from INS-LANano was observed than blank LANano, which implies that loading of insulin to the blank formulation improved the cytocompatibility of the formulation. Permeability experiment revealed that LANano at its nontoxic concentrations could significantly improve the transport of insulin across Calu-3 monolayer than that of INS-SEN and INS-SOL. Previously, the Calu-3 cell line was used to evaluate the cellular uptake of insulin-encapsulated glycopolymer nanoparticles and the effective transport of insulin into cells than that of the control formulation was observed [29]. Polycationic materials, such as poly-l-arginine, have been shown to increase the paracellular permeability of a model hydrophilic macromolecule fluorescein isothiocyanate labeled dextran (FD-4) across Caco-2 cell monolayers by dispersion of TJ proteins into the cytoplasm [30,31]. Further investigation of the underlying mechanism of TJ disassembly revealed that poly-l-arginine induced the transient internalization of TJ proteins via clathrin-mediated endocytosis, thereby enhancing the paracellular permeability of FD-4 [32]. In our study, a significantly higher permeability of insulin by INS-SEN was also observed, which can be attributed to the presence of Capmul MCM as a formulation component of the SEN [33] that also acts as a permeation enhancer. SEN promotes enhanced permeation but binding of insulin to SEN via Arginine may also facilitate the transport.

Several technologies have been developed with absorption enhancers to improve the in vivo relative bioavailability of insulin via the nasal route [34,35]. An arginine-rich cell-penetrating peptide, L-penetratin, was administered to healthy anesthetized rats in 0.2–2 mM concentration with 1 IU/kg insulin and the resulting relative bioavailability of insulin was in the range of 15.1 ± 4.7% to 50.7 ± 6.8% [36]. Cationic polysaccharide chitosan was used as a permeation enhancer for insulin in a powder formulation and upon administration to non-anesthetized healthy sheep at a dose of 128 IU/sheep, they produced a BA of 17.0 ± 6.6% [37]. However, chitosan and other carbohydrate polymers are non-biodegradable while LA-Nano ingredients are biodegradable. A lyophilized insulin-dimethyl-β-cyclodextrin powder formulation achieved a 12.9 ± 4% of absolute bioavailability when administered at a 4 IU/rabbit dose on healthy anesthetized rabbits [38]. We have used non-anesthetized rats in our animal study. It was found in an investigation, designed to observe the effect of anesthesia on intranasal absorption of insulin co-administered with sodium deoxycholate in rats, that absorption of insulin varied with the use of different anesthetic agents whereas rats under no anesthesia showed comparatively lower absorption than that of the anesthetized rats. The proposed reason was the variable degree of mucociliary clearance of formulation by different anesthetic agents [39]. Afrezza^®^ is the only non-invasive insulin product in the market. Thus, to understand the translational potential, a table comparing the marketed formulation Afrezza^®^ with our product is a provided (Table 6).

## 5. Conclusions

Electrostatic binding between negatively charged α-helix of insulin and arginine exposed positively charge nanoglobules was successfully characterized. Permeability enhancing effect of lipids especially medium chain monoglyceride and INS-LAnano interaction resulted in high insulin permeability in vitro and in vivo. Insulin-loaded arginine-coated nanoglobules significantly enhanced the absorption of insulin across monolayers with significantly higher relative bioavailability than insulin solution. Therefore, INS-LANano has the potential to deliver human insulin effectively via the nasal route.

## Figures and Tables

**Table 1:** Size and Zeta Potential of SEN and LANano.

Formulation	Zeta (mV)	Size (nm)	PDI
SEN (Water)	−0.241 ± 0.942	26.84 ± 0.19	0.051 ± 0.018
LANano (Water)	+13.10 ± 0.794	14.04 ± 0.12	0.383 ± 0.001
LANano (PBS)	+3.040 ± 0.335	22.70 ± 0.60	0.134 ± 0.015

Mean ± SD, N = 3.

**Table 2:** Size and Zeta Potential of LANano with Different Concentrations of Insulin.

Conc. of Insulin (µg/mL)	LAE to Insulin Ratio	Zeta (mV)	Size (nm)	PDI
100	1:0.1	+1.341 ± 0.941	21.74 ± 0.07	0.022 ± 0.016
300	1:0.3	+0.559 ± 0.497	21.69 ± 0.19	0.042 ± 0.014
500	1:0.5	+0.118 ± 0.095	23.38 ± 0.62	0.089 ± 0.013
700	1:0.7	+0.281 ± 0.089	21.78 ± 0.42	0.077 ± 0.023
900	1:0.9	−0.081 ± 0.153	22.87 ± 0.47	0.068 ± 0.021
1000	1:1.0	−0.375 ± 0.540	23.38 ± 0.40	0.080 ± 0.005

Mean ± SD, N = 3.

**Table 3:** α-Helix Content of Insulin.

Insulin to LAE Ratio	Peak/Valley Wavelength (nm)	α-Helix Content (%)
1 to 0.0	198.5/208.5, 223.0	42.0
1 to 1.0	198.5/209.0, 225.0	35.7
1 to 2.5	198.0/209.5, 224.0	35.5
1 to 5.0	199.0/209.5, 221.5	24.9
1 to 7.5	196.5/209.0, 224.0	22.9
1 to 10.0	199.5/215.5, 224.0	13.4

**Table 4:** Recovery (%) of ultrafiltered insulin in Receiver Chamber.

Formulations	Recovery (%)
INS-SOL	91.5 ± 1.5
INS-SEN	86.4 ± 3.6
INS-LANano	53.6 ± 0.1 *

Mean ± SD, N = 3. *: Significantly lower than INS-SEN (*p* < 0.001).

**Table 5:** Pharmacokinetic Parameters.

Groups	Dose (IU/kg)	C_max_ (mU/L)	T_max_ (min)	AUC _0–120 min_ (mU/L × h)	BA (%)
INS-SOL (S.C.)	1	11.1 ± 1.1	36.7 ± 12.0	16.2 ± 2.6	100
INS-LANano (I.N.)	2	14.3 ± 2.7 *	12.5 ± 2.2	11.3 ± 0.9	23.3 ± 1.8 *^,^**
INS-SEN (I.N.)	2	8.8 ± 2.6	10.0 ± 0.0	7.7 ± 0.3	15.8 ± 0.6 *
INS-SOL (I.N.)	2	4.1 ± 0.2	13.3 ± 3.3	3.4 ± 0.4	6.9 ± 0.8

Cmax: maximum plasma insulin concentration; Tmax: time to reach Cmax; AUC: area under the insulin concentration-time curve; I.N.: intranasal administration; SC: subcutaneous administration; BA: relative bioavailability compared with SC; Each value represents the mean ± SEM (n = 3–4); *: Significantly higher than INS-SOL (I.N.) **: Significantly higher than INS-SEN (I.N.); (*p* < 0.05).

**Table 6:** Comparison of Afrezza and INS-LANano.

	Criteria	Afrezza^®^	INS-LANano
1	Formulation technology	It is a dry powder formulation where recombinant human insulin is adsorbed onto fumaryl diketopiperazine particles as carrier to deliver insulin via the pulmonary route [4]	It is an aqueous formulation where recombinant human insulin is bound to the surface of Arginine-coated nanoglobules as carrier to deliver insulin via nasal route
2	Comparison of Pharmacokinetic Profile	In non-smoking healthy volunteers, the BA (compared to subcutaneous) is about 24.6%, 22.9% and 20.6% and the Tmax is about 12 min, 15 min and 17 min at 25 U, 50 U and 100 U doses, respectively [20]	In diabetic rats the BA and Tmax is about 23.3% and 12.5 min
3	Adverse Effects	Acute bronchospasm in patients with asthma and COPD Decline in pulmonary function over time Cough and throat pain or irritation Not recommended in patients with active lung cancer, have a history or are at risk of lung cancer [4]	Further studies needed to understand; Toxicity to nasal mucosa Physiological and protective function of nasal defensive enzyme Bioavailability on long term use Nasal dryness, irritation, sneezing, congestion [21]
4	Effect of Smoking	Not recommended for smoker patients [4]	Further studies needed to understand it. For Miacalcin Nasal Spray, smoking did not show a contributory effect on the occurrence of nasal adverse reactions)
5	Complexity of delivery device	Complex	Simple

## Data Availability

No new data were created or analyzed in this study. Data sharing is not applicable to this article.

## Abbreviations

INS-LANano, insulin-loaded arginine-coated self-emulsifying nanoglobule; LANano, arginine-coated self-emulsifying nanoglobule; INS-SEN, insulin-loaded self-emulsified nanoemulsion; SEN, self-emulsified nanoemulsion; INS-SOL, insulin solution; BA, Relative Bioavailability.
